# Strategies to Mitigate Chemotherapy and Radiation Toxicities That Affect Eating

**DOI:** 10.3390/nu13124397

**Published:** 2021-12-08

**Authors:** Peter M. Anderson, Stefanie M. Thomas, Shauna Sartoski, Jacob G. Scott, Kaitlin Sobilo, Sara Bewley, Laura K. Salvador, Maritza Salazar-Abshire

**Affiliations:** 1 Pediatric Hematology/Oncology and Bone Marrow Transplant, Cleveland Clinic Children’s, Cleveland, OH 44195, USA; thomass29@ccf.org (S.M.T.); sartoss@ccf.org (S.S.); sobilok@ccf.org (K.S.); bewleys@ccf.org (S.B.); 2 Taussig Cancer Institute, Cleveland Clinic, Cleveland, OH 44195, USA; scottj10@ccf.org; 3 Department of Nursing, Cleveland Clinic, Cleveland, OH 44195, USA; 4 Department of Radiation Oncology, Lerner Research Institute, Cleveland Clinic, Cleveland, OH 44195, USA; 5 Peds Nutritional Services, Cleveland Clinic, Cleveland, OH 44195, USA; 6 Department of Pediatrics, MD Anderson Cancer Center, Houston, TX 77030, USA; lekaye@mdanderson.org (L.K.S.); msalaza@mdanderson.org (M.S.-A.); 7 Department of Nursing Education, MD Anderson Cancer Center, Houston, TX 77030, USA

**Keywords:** outpatient chemotherapy, nausea, anti-emetics, catabolic state, sarcopenia, sterotactic body radiotherapy (SBRT), therapeutic alliance, cancer treatment side effects

## Abstract

Background: Cancer and its therapy is commonly associated with a variety of side effects that impact eating behaviors that reduce nutritional intake. This review will outline potential causes of chemotherapy and radiation damage as well as approaches for the amelioration of the side effects of cancer during therapy. Methods: Information for clinicians, patients, and their caregivers about toxicity mitigation including nausea reduction, damage to epithelial structures such as skin and mucosa, organ toxicity, and education is reviewed. Results: How to anticipate, reduce, and prevent some toxicities encountered during chemotherapy and radiation is detailed with the goal to improve eating behaviors. Strategies for health care professionals, caregivers, and patients to consider include (a) the reduction in nausea and vomiting, (b) decreasing damage to the mucosa, (c) avoiding a catabolic state and muscle wasting (sarcopenia), and (d) developing therapeutic alliances with patients, caregivers, and oncologists. Conclusions: Although the reduction of side effects involves anticipatory guidance and proactive team effort (e.g., forward observation, electronic interactions, patient reported outcomes), toxicity reduction can be satisfying for not only the patient, but everyone involved in cancer care.

## 1. Introduction

Cancer remains a major public health problem in North America [1,2,3] and world-wide with >10 million deaths/year attributable to cancer [4]. What is feared by both patients and their caregivers is toxic therapy that may or may not be effective and makes eating a difficult and unpleasant experience especially when it is needed most. The proliferation of internet information (and some misinformation) along with very common use of dietary supplements shows how patient and caregivers perceive the life-or-death nature of cancer not only as very serious, but also quite worthy of extra time and effort to improve well-being. Furthermore, patient/caregiver time away from work or school coupled with high costs of cancer therapy also make cancer emotionally and financially toxic [5,6,7,8,9,10,11,12,13]. 

State-of-the-art cancer therapy utilizes advanced surgery and complex systemic treatments (chemotherapy combinations and/or radiation) for this often life-threatening condition. Organizations such as the Multinational Association for Supportive Care in Cancer (MASCC), the European Society of Clinical Oncology (ESMO), the American Society of Clinical Oncology (ASCO), and the National Comprehensive Cancer Network (NCCN) have provided guidelines to help with information and education about chemotherapy and radiation induced mucositis, nausea and vomiting [14,15,16,17,18,19,20,21].

**Figure 1:**
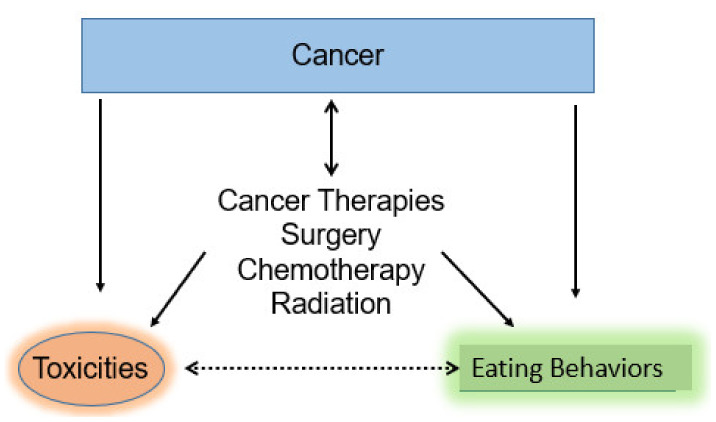
Bidirectional and complex nature of the three main cancer treatment modalities (surgery, chemotherapy, and radiation) on toxicities and eating behaviors. Complex interactions of therapy affect quantity and quality of nutrient intake and toxicities encountered when attempting to reduce or eliminate cancer cells.

Weighing indications, risks, and alternatives to achieve the highest benefit with lowest toxicity (i.e., high therapeutic index) is the current dynamic of cancer treatment for each person. Individual differences in cancer therapy tolerance are especially broad for the very young (infants and toddlers) and older (geriatric) persons. Chemotherapy drugs (Table 1) and radiation (Table 2) affect cancer cells and normal tissue differently. How cancer and its therapy can affect eating behaviors and contribute to toxicity in a complex, bidirectional manner is illustrated in Figure 1. Patient education about chemotherapy drugs and radiation often includes long and sometimes very confusing lists of potential side effects. A more organized approach is to review dose, schedule, drug combinations, and/or radiation with overlapping and non-overlapping toxicities in the context of common, less common and rare, as well as immediate, delayed, and long-lasting toxicity to normal tissues. Specialized oncology pharmacists coupled with tools such as hand-outs found on chemocare.com and Children’s Oncology Group can provide patients and families a manner to organize this highly specialized information. 

**Table 1:** Chemotherapy regimen variables to kill cancer cells with better normal tissue tolerance.

Chemotherapy Regimen	Effect on Normal Tissue	Tumor Versus Normal Tissue Consideration(s)
Dose	Side effects against a normal tissue (for example, production of platelets by bone marrow) are dose-limiting	An optimal biologic dose (OBD) instead of the maximally tolerated dose (MTD) may facilitate normal tissue healing. An area under the curve (AUC) strategy with oral dosing or continuous infusion can decrease toxicity of some drugs (e.g., cyclophosphamide or ifosfamide [22,23])
Mechanisms of action against dividing cells	Marrow, mouth, esophagus, intestines, and skin are easily damaged by chemotherapy	Chemotherapy guidelines should allow adequate tissue recovery before administration of the next cycle allowing for improvement in blood counts, mucositis, diarrhea, and skin)
Biodistribution	Oral mucosa and skin blood flow are temperature dependent	Oral cryotherapy can reduce mucositis [24,25,26,27] Cold packs may decrease hand–foot erythroderma
Drug metabolism	Elimination and inactivation of chemotherapy drugs vary between tissues and persons	Dose adjustment if excessive toxicity is seen Facilitate detoxification by normal cells (e.g., improve glutathione [28,29,30])
Protective drugs	Can reduce damage to normal tissues to mitigate or avoid significant short-term or long-term side effects	Mesna to protect the bladder from acrolein metabolite after cyclophosphamide or ifosfamide Dexrazoxane to protect heart from doxorubicin Dexamethasone to prevent taxane reactions Leucovorin to rescue from methotrexate
Drug combinations	Combinations of chemotherapy drugs can be more toxic to organs and tissue than a single agent	Chemotherapy combinations with non-overlapping toxicities and alternating regimens to achieve less toxicity are often used

Cumulative organ toxicity can occur with repeated cycles of chemotherapy (e.g., cochlea [31], kidney, heart, lung, “chemobrain”). If an oncology team orders the next chemotherapy cycle before normal marrow cells recover (for example, inadequate red cell, white cell, or platelet recovery), the next cycle may require longer recovery time because the lowest point (nadir) becomes lower. However, if an oncology team waits too long to start the next chemotherapy cycle, cancer cells may proliferate or spread while waiting for normal tissue recovery. Thus, each chemotherapy cycle should kill more cancer cells than are able grow back while allowing normal tissues to heal between cycles.

**Table 2:** Radiation damage associations: variables to damage tumor with less normal tissue side effects.

Damage Association	Variable	Tumor Versus Normal Tissue Consideration(s)
Fraction dose size	Amount of radiation energy per dose	Larger fractions are biologically more effective against tumors than normal cells
Schedule	One-time, daily for 1 week, or daily (e.g., M–F) for 3 to 5 weeks	Time between radiation doses allows both tumor and normal tissue repair
Tumor radiosensitivity	Some cancers (e.g., Wilms tumor, lymphomas) are very radiosensitive. Other cancers (e.g., carcinomas, brain tumors, sarcomas, metastases) can be more difficult to kill with radiation.	Smaller total dose is needed to treat some tumors with curative intent. If a tumor is relatively radioresistant, a combination of chemotherapy and radiation may work better against tumor cells [32,33,34]
Radiation particle	Photons and electrons have less energy than protons and alpha particles. More energy results in hard to repair double strand DNA breaks in cancer cells	Choice of the type of radiation often depends on normal tissue nearby as well as the dose needed to treat
Precision of radiation treatment plan	Stereotactic body radiotherapy (SBRT) sterotactic radiosurgery (SRS) and proton radiotherapy plans are very precise. These require not only expensive radiation machines, but a highly specialized radiation physicist and oncologist time and effort for each individualized treatment plan	Palliative radiation plans are less precise and use lower doses for rapid treatment planning to reduce pain with acceptable (low) damage to nearby tissue. Image guidance provides more precise radiation treatment plans (more to tumor and less to normal tissue) Very precise SBRT, SRS, or proton plans may take 1–2 weeks before the patient can start radiotherapy in a manner that treats tumor and minimizes radiation to nearby normal tissue

Although oncology is a very scientific and evidence-based discipline with hundreds of thousands of studies for general and specific questions and common-sense consensus guidelines by experts to guide clinicians and patients [3,21], our ability to ameliorate toxicities and predict survival outcomes for an individual is limited. Estimates often are “guestimates” and vary with time and experience. Medical professionals and cancer patients alike believe toxicities are inevitable and general amelioration techniques are often overlooked. Difficulty eating and malnutrition during cancer therapy are common problems that can be assessed with many tools [35,36,37,38,39,40,41,42,43,44,45,46,47,48,49,50,51]. The challenge is to have an acceptable quality and quantity of nutrient intake with minimal toxicity without compromising effective cancer treatment. 

Complex and bidirectional interactions occur between cancer, therapy, toxicities, and eating behaviors (Figure 1). This article will review some mitigation strategies to organize patients and caregivers during virtual visits [52], hospitalizations, and outpatient clinic discussions with oncologists, nurse practitioners (NP), physician assistants (PA), navigators, pharmacists, nurses, and dietitians. Nursing educators and oncology navigators are relatively new positions in the oncology team who can multiply the effectiveness of education efforts by helping not only individual patients, but also educate inpatient and outpatient oncology nurses. Additionally, quality and continuous improvement efforts can facilitate creating systems in oncology clinics and hospitals to foster health literacy and to adapt and adopt patient and family-centered best practices [53,54,55,56,57]. 

## 2. Review of Strategies to Improve Eating Behaviors While Receiving Chemotherapy and/or Radiation

One of the most common questions of cancer patients and caregivers is “what should I eat or drink to improve my situation?” [58] This topic is very important but sometimes relatively neglected because of many time-consuming tasks associated with accurately prescribing chemotherapeutics and radiation therapy accurately and on time [58,59,60,61,62,63,64,65]. Fortunately, dietitians with oncology experience can provide tools and information to patients regarding food choices, eating behavior, and issues including poor oral intake and intermittent fasting when patients get chemotherapy and/or radiation [66,67,68]. Not only infusion nurses, but also oncology educators and navigators can use patient and caregiver feedback to increase dietitian consultation for cancer patients. Malnutrition in cancer patients is a common problem. There are many indices and tools to define malnutrition, cachexia, and sarcopenia [35,36,37,38,39,40,41,42,43,44,45,46,47,48,49,50,51,62,69,70,71,72,73,74,75,76,77]. Although weight loss from the time of diagnosis is one measure, the quantity and quality of food in the diet and information from the patient generated subjective global assessment (PG-SGA) tool can provide more specific information [35,36,45,51,62,66,69,70,71,72,73,78,79,80]. 

**Figure 2:**
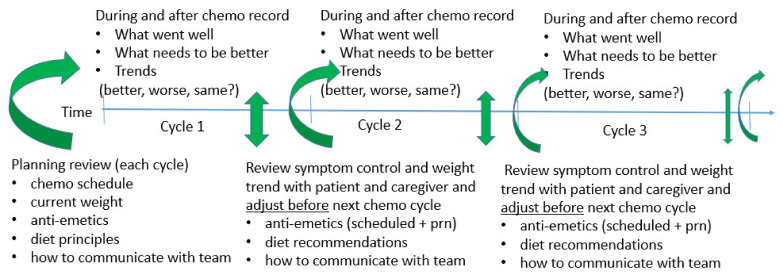
Proactive, adaptive, eclectic, and flexible approach to continuously improving symptoms and eating so each chemotherapy cycle gets better, and daily activities become more normal with fewer adjustments needed.

Nevertheless, weight stability, weight loss, or weight gain is what the oncologist, oncology NP or PA needs when calculating and confirming chemotherapy drug dosage and is imperative for safety prior to prescribing chemotherapy. Typically, unless a >10% change is noted, no changes are required in chemotherapy dose. However, 5–10% body weight loss usually requires redoubled efforts to improve weight. If weight gain is real, which sometimes happens in children during leukemia chemotherapy, dose is increased. If a gain is “artificial” (e.g., fluid retention or ascites), chemotherapy doses are not increased. If there is >10% weight loss, then additional nutrition support is given via enteral feeding device (e.g., NG or G-tube), or total parenteral nutrition (TPN) intravenously if unable to feed enterally. In this circumstance, chemotherapy dosage is usually decreased. Higher level nutritional intervention can also reduce or avoid chronic nausea unrelated to chemotherapy administration that is associated with superior mesenteric artery syndrome with omental and mesenteric fat loss from chronic inadequate nutrients. A reasonable goal of the cancer patient and family should be to optimize nutrition with less effort so the positive social aspects of meals together and nutrient intake are enjoyable (Figure 2). Quality metrics in the hospital and oncology clinic can assist programs to achieve less toxicity and better resource utilization both routinely [81] and when efforts like TPN [49,82,83] and hospitalization [7,12] are utilized.

### 2.1. Nausea as a Source of Poor Appetite: Approaches to Reduce Nausea

Unfortunately, a common side effect of chemotherapy and radiation is nausea and/or vomiting (N/V). In the past 25 years, major advances in nausea and vomiting reduction from chemotherapy and/or radiation therapy have improved this situation. These include the following: common use of selective serotonin receptor (5-HT3 also known as 5HT) antagonists for immediate N/V, neurokinin receptor antagonists (aprepitant and fosaprepitant) for delayed nausea, and the recognition of corticosteroids and olanzapine in front-line anti-emetic regimens for chemotherapy and radiation [19,21,84,85,86,87,88]. Although N/V from chemotherapy and/or radiation remains common, there are many effective strategies to ameliorate N/V and improve eating behaviors during chemotherapy and radiation as detailed in Table 3. 

**Table 3:** Causes of nausea and vomiting (N/V), reduction agents, and mitigation strategy issues.

Cancer Therapy N/V Cause and Associations	Anti-Emetic Agents and Mechanisms of Action: Generic Name (Brand Name)	Strategy, Some Practical Considerations, and References
Immediate N/V from chemotherapy agents: chemoreceptor trigger zone	Selective serotonin receptor (5HT) antagonists: Ondansetron (Zofran) Granisetron (Kytril, Sancuso) Palonosetron (Aloxi)	MASCC + ASCO anti-emetic guidelines for chemotherapy and radiation [19,21]; ondansetron has more drug interactions. Granisetron is an oral or transdermal patch. Palonosetron is IV, has fewer drug interactions and longest half-life [84,86,87].
Dysmotility	Dopamine agonists: Metoclopramide (Reglan) Prochlorperazine (Compazine)	Use with caution in children. Dopamine agonists can cause extrapyramidal symptoms including dystonic reactions.
Inflammation	Corticosteroids act on both immune cells and tumor microenvironment: Dexamethasone (Decadron) Methyprednisolone (Medrol) Prednisone Hydrocortisone	Excellent for 1–7 days; high-doses can increase appetite and eating, but cannot use long term because of chronic issues, including: infection, appearance, skin thinning, blood pressure, diabetes, osteoporosis and avascular necrosis of shoulder, hip, and knee joints
Delayed N/V: Many hours to days after starting chemotherapy	Neurokinin receptor antagonists: Aprepitant (Emend) oral Fosaprepitant (Ivemend in EU)	Few drug interactions; especially effective with cisplatin. Can give fosaprepitaint intravenously on days 1 and 4 of each 5-day cycle
Anticipatory N/V	Help change context of N/V Lorazepam (Ativan) Diphenhydramine (Benadryl)	Change environment or routine Take meds before coming to clinic Oral or IV possible; some sedation is associated with these agents which may be an undesirable side effect especially if it impedes nutritional intake
Sleep deprevation	Promote routine sleep Melatonin Olanzapine (Zyprexa) Diphenhydramine (Benadryl)	At bedtime melatonin 2–10 mg per day olanzapine may also help mood sedating
Decreased appetite	Central acting Tetrahydrocannabinol (THC) Medroxyprogesterone (Megace)	Oral before meals THC also has antiemetic activity Both pills and liquid available
Motion sickness	Scopolamine (Transderm Scop)	Patch for 3 days
Multiple causes	Anti-emetic combinations often more effective than single agents	MASCC, ESMO, ASCO, and NCCN guidelines [14,15,16,17,18,19,20,21]

### 2.2. Proactive Approaches to Toxicity Reduction for Better Eating Behaviors (Forward Observation)

Figure 2 illustrates a proactive and adaptive approach towards nausea and vomiting reduction during sequential chemotherapy cycles. This involves shared decision making and understanding chemotherapy dosing and schedule using current weight trends, side-effect profiles, and toxicity prevention strategies through adaptation and continuous improvement. Continuous improvement requires the patient and caregivers providing the oncology team with high quality information about what was effective during a chemotherapy cycle and what could be improved in the next cycle. This information can then guide adjustments with each cycle such as the dose or schedule, and the addition or substitution of different anti-emetic agents, and dietary modifications. An adaptable and eclectic approach avoids “running the same play” resulting in the same or worse toxicity and possibly having increasingly worse eating behaviors. The goal is to help the patient and family avoid repetitive and/or cumulative issues with N/V and reduced appetite during chemotherapy so that eating becomes predictably better and enjoyable. Meals are then not major issues, but rather a source of companionship, social interaction, and improved well-being. 

### 2.3. Mucosal and Skin Injury

Another problem from chemotherapy and radiation is damage to the mucosal lining cells of the mouth, esophagus, stomach, intestines, and rectum (mucositis) [14,16,17,18,20,89,90,91,92,93,94,95]. The reduction in stomatitis (mouth sores), esophagitis, enteritis, and/or proctitis (rectal pain) caused by chemotherapy and radiation will improve eating behavior and nutrient choices during cancer therapy. Mucositis is a common problem and affects a variety of tissues not only from the toxicity of chemotherapy drugs, but also from radiation affecting tissue near the tumor target, too. Mucosal injury, especially to the mouth and GI tract, is often painful but transient. 

Unfortunately, sometimes long-lasting toxicities occur (e.g., neuropathy, cardiotoxicity, esophageal or intestinal stricture/dysfunction long after completion of chemotherapy and/or radiation), therefore, efforts to rate treatment intensity and to mitigate cancer therapy toxicities are important to long-term health [96]. Many mucositis prevention strategies and treatments have been evaluated and have been summarized by MASCC/ISOO guidelines [14,16,17,18,20,89]. Table 4 lists specific agents and mucositis reduction strategies. Table 5 details agents and causes that contribute to epithelial toxicities of chemotherapy and/or radiation and some mitigation strategies. Physical therapy (PT) and occupational therapy (OT) can be helpful in maintaining mobility and developing strategies to accomplish activities of daily living including eating as part of education about long-term health in cancer patients.

**Table 4:** Mouth sores, esophagitis, and enteritis: agents, injury type, and reduction during cancer therapy.

Agent	Type of Injury	Reduction Strategies
Melphalan	High dose alkylator (peak effect)	Cryotherapy (ice chips) [24,25,26,27] Keratinocyte growth factor (palifermin) [97,98,99,100,101]
Gemcitabine	Cytotoxic injury of mucosal cells	A 30 min infusion is less toxic than a 90 min infusion. Avoid daily dosing. Often used weekly × 2 weeks (day 1, day 8) and then 1 week off
5-Flurouracil	Cytotoxic injury to mouth	Cryotherapy; change schedule
Doxorubicin	Cytotoxic injury, radiation recall	Use dexrazoxane, then a short infusion of doxorubicin, instead of continuous infusion to reduce heart injury; to reduce mucositis use glutamine + trehalose (Healios, [89])
Mammalian target of rapamycin (mTOR) inhibitors	Cytotoxic injury by sirolimus, temsirolimus, everolimus	Follow blood levels and adjust dose; use glutamine + trehalose [89]
Vascular endothelial growth factor (VEGF) inhibitors: bevacizumab, pazopanib, cabozantinib, regorafenib	Less of an ability to form new blood vessels: wounds and radiation injured tissues heal slowly	Dose reduction or drug holiday utilizing intermittent dosing of oral agents (e.g., three weeks on then one week off)
Irinotecan	Active metabolite of irinotecan in the intestinal lumen, SN-38, causes intestinal mucosa injury	Reduction of immediate cramping, diarrhea, and GI upset: loperamide +/− atropine. For intermediate and delayed diarrhea octreotide and/or glutamine + trehalose (Healios [89]). If eating solid food is difficult, liquid nutritional supplements can be helpful
Yeast	Broad spectrum antibiotics reduce normal bacterial flora	Anti-fungal antibiotics, yogurt, kefir; limit anti-bacterial antibiotics unless suspected or known infection(s)
Radiation	Harms rapidly dividing cells in the renewing tissues (crypts) lining the mouth, esophagus, intestines, rectum	Protein to heal and/or glutamine + trehalose; review radiation plan (dose/schedule) to allow some healing (e.g., weekends off); boost radiation to tumor volume only. Keep skin and mucosal surfaces clean. If whole abdominal radiation therapy (WART): g-tube for additional enteral nutrition

Radiation recall is a rash after chemotherapy resembling a sunburn only in skin previous exposed to radiation (in-field). Radiation recall rash can also give clues about damage to nearby structures beneath the skin that are also in the radiation field (e.g., mouth, esophagus, stomach, intestines) that can affect eating. It is possible to decrease skin toxicity with creams such as Eucerin, Glucan-Pro or topical corticosteroids. Furthermore, less skin toxicity can improve sense of well-being and feeling “normal”, thereby facilitating more exercise and activity and improving eating behavior. Table 5 details some strategies to reduce epithelial and skin toxicity and to promote wound healing.

**Table 5:** Reducing chemotherapy and radiation damage in epithelial tissues (skin and mucosa).

Cause of Damage	Type of Injury and Consequence(s)	Reduction Strategies
Radiation	Single and double-stranded DNA breaks: cell death	Anticipatory guidance that damage may last longer than radiation treatment. Glutamine seems protective for some tissues, especially intestines [30,89,102,103,104,105]
Radiotherapy (RT) or surgery and VEGF inhibitors	Tissue may heal slowly after VEGF inhibitors due to decrease in vasculature	Use the oral tyrosine kinase inhibitor (TKI) with shorter half-lives so if there are symptoms, these can be stopped, and then restarted sooner (when the wound or injury is improved)
Corticosteroids: prednisone methylprednisolone dexamethasone	Thinning of skin Delayed wound healing Increased risk of infections	Use short 1–5 day pulses. If prolonged use, then taper to hydrocortisone in physiologic doses (e.g., 15 to 20 mg am and 10 mg pm)
Opiates	Slower GI motility causes nausea, constipation, hard stool with rectal fissures and perirectal inflammation may recur with each cycle of chemotherapy when patient may experience tissue damage and subsequent infection due to low neutrophil counts	Increase liquid in diet and physical activity as tolerated for patient. When appropriate consults to PT/OT. Kefir, stool softeners such as docusate, lactulose, polyethylene glycol powder 3350 (MiraLax), and senna can be helpful. Use of a hand-held shower and baby wipes or cotton-balls with lotion may clean rectal tissues with less damage
Total parenteral nutrition (TPN)	Prolonged use is associated with villous atrophy of intestine lining and liver toxicity	Some trophic enteral nutrition is needed to increase absorptive surface of intestines and to avoid liver toxicity
Abdominal surgery then RT to abdomen especially whole abdominal radiotherapy (WART)	Less GI motility post-op delays eating increases radiation enteritis. Effects of radiation can cause future small bowel obstruction	Gastrostomy tube (g-tube) to facilitate hospital discharge and eating sooner with better enteral nutrition before, during, and after whole abdominal radiotherapy (WART). PT/OT and dietitian consults
Head and neck RT C-spine or T-spine RT (with or without concurrent chemotherapy)	Often associated with severe mucositis including mouth sores (stomatitis) and oropharyngeal and esophageal mucositis	MASCC guidelines [15,16,17,20]. Pain medicine before eating and OT consult; glutamine + trehaose suspension (Healios) swish 10 s then spit or swallow [89]
High-dose chemotherapy +/− RT (preparative regimens for bone marrow transplant (BMT)	Very high incidence of mucositis. Extra inflammation may predispose to graft versus host disease (skin, mouth, and GI toxicities). Toxicities can cause eating and activity issues	Palifermin [97] and/or a very high level of supportive care (TPN, G-tube or NG tube) [27,85,90,106,107,108]. PT/OT and education to maintain “trophic” enteral can be helpful. MASCC guidelines [15,16,17,20]

Hematologic toxicity from chemotherapy and radiation can adversely affect nutrient intake. Anemia caused by decreased production of red blood cells by the bone marrow as a direct effect of chemotherapy and radiation can cause fatigue. Sometimes iron deficiency from blood loss or inadequate iron intake is seen even before anemia becomes apparent. Iron deficiency can also cause a protein losing enteropathy which makes iron absorption more difficult. Fortunately, iron depletion or deficiency can be corrected easily and quickly (1–3 days) using intravenous iron supplementation (e.g., iron sucrose, Venofer). Red cell or platelet transfusions are sometimes needed to support patients with chemotherapy associated severe anemia or low platelets (thrombocytopenia) or during radiation to keep tissue oxygenation high and decrease risk of bleeding. Patients will often feel much better and have less fatigue if transfused at a medium low hemoglobin (e.g., Hb 8) and/or downward trending instead of waiting until anemia becomes quite severe.

Discussion of indications, risks, and alternatives and “using patient as their own control” to decide on transfusion or growth factor thresholds are common practices in oncology. Growth factors can increase the neutrophil type of white cell numbers, decrease infection risk and keep chemotherapy cycles on time. Granulocyte colony stimulating factor (G-CSF) is commonly used in North America and Europe. Growth factors for red cell production (e.g., erythropoietin or darbopoietin) and platelet production (romiplostim or eltrombopag) are used less often, but also can be effective. Better neutrophil counts are associated with better mucosal healing, too. Finally, lymphopenia, a common side effect of chemotherapy and radiation is associated with not only increased infection risks (e.g., pneumocystis pneumonia), but also decreased survival [109]. More rapid recovery of lymphocytes is associated with improved survival [109,110,111,112]. Since the metabolic fuel for lymphocytes is glutamine, a diet with adequate protein may be beneficial and outweigh any deleterious effects of glutamine supplementation such as an energy source of tumor cells [89]. Since glutamine is always the highest amino acid in the blood and subject to homeostatic regulation (muscle will breakdown if there is not enough protein in the diet) to maintain plasma glutamine levels, glutamine and or protein supplementation should be considered as a means to provide local and systemic anabolic effects.

### 2.4. Deconditioning and Fatigue

Deconditioning associated with the loss of muscle mass (sarcopenia) is another potential consequence of chemotherapy and radiation toxicity. First, voluntary or involuntary confinement to a hospital room can severely reduce activity. Well-meaning activity limits by caregivers to limit falls can result in fewer steps and a profoundly sedentary life-style for cancer patients (e.g., less activities of daily living such as shopping, dining out, mowing the lawn, washing clothes, cleaning, doing the dishes for outpatients). Once deconditioning occurs, routine activities become increasingly difficult. To achieve a Karnofsky performance scale (KPS) level of 100%, activities of daily living including predictable eating behaviors and walking more should be actively encouraged. Sometimes a step counter device (e.g., Fit-bit or Apple watch) can provide meaningful and accurate feedback about activity level and help avoid deconditioning. Ensuring that consultations are made with our allied health partners (such as physical therapy, occupational therapy and when working with pediatrics to child life therapy) also serves to promote regular physical activity and avoid deconditioning that so often accompanies chemotherapy and radiation therapy toxicity. Consultations to our behavioral science partners such as psychology and psychiatry should also be made to combat fatigue associated with depressed mood that may result from confinement to the hospital, cancer treatment, or from the diagnosis of cancer in an individual.

If possible, cancer patients should discuss advantages and limitations of outpatient versus inpatient chemotherapy regimens with the oncology team; outpatient chemotherapy often results in better eating behavior and more activity with less deconditioning and fatigue. Sarcopenia (less muscle mass) is probably related to both deconditioning (if you do not use it, you lose it) as well as inadequate calories and protein. Less sarcopenia has been associated with fewer fevers associated with neutropenia and improved survival [75,76,77,113].

### 2.5. Improved Information

When professionals make decisions, the variability of skills needed, and tasks required, seems impossibly complex and full of noise. However, when patients and caregivers make use of professionals and networks of people with many different and complementary skill sets, this eclectic approach can help to get the best information from a variety of sources. The author has termed this activity developing therapeutic alliances [52,114]. Quality specialists, nurse educators, oncology navigators, nurse practitioners, physician assistants, and dietitians with oncology experience are especially skilled at facilitating therapeutic alliances and family-centered care [13,53,54,55,56,96,114,115]. Table 6 illustrates some aspects of developing therapeutic alliances to anticipate and ameliorate chemotherapy and radiation toxicity and improve the journey of receiving cancer therapy.

## 3. Discussion

Chemotherapy and radiation toxicity reduction should result in better eating and less sarcopenia [47,74,75,76,77,78,113,116,117,118]. This complex and often bidirectional interaction (Figure 1) may also translate into fewer episodes of fever and neutropenia and improved survival [75,76,77]. Lymphopenia (low lymphocyte counts) is associated with radiation and worse survival [109]. Better eating may also improve immune function by supplying glutamine for lymphocytes and better immune function [119,120,121,122]. Finally, nutrient intake may have some cancer prevention properties [123,124] and help with glutathione production to ameliorate toxicity [28,29] and to reduce some chemotherapy and radiation associated toxicity [30,103,104,105,125,126,127]. Since cancer is more prevalent in older adults, the problem of increased toxicity associated with poor eating in sarcopenic, frail, older patients with co-morbidities and polypharmacy can be challenging [74,77,118,128,129,130,131,132]. Use of the Geriatric Nutrition Risk Index (GNRI) or Mini-Nutritional Assessment (MNA) tools [133,134,135] and patient reported outcomes (PRO) such as the PG-SGA [69,70,71,72,73,136,137,138,139,140] could help to predict risk and trigger more timely interventions to address eating behaviors, malnutrition, cachexia, and sarcopenia in this particularly vulnerable, high-risk population. A recent study showed a proactive team approach which included an oncologist, NP, social worker, PT/OT, pharmacist, and nutritionist resulted in the significant reduction of grade 3 or higher toxicities in older adults [141].

Obesity has become increasingly prevalent and is associated with the increased cancer risk and worse cancer outcomes [142,143,144,145,146,147,148,149,150]. Increased BMI can adversely affect response to agents such as VEGF inhibitors [146], endocrine therapy in breast cancer [144], hematologic chemotherapy toxicity in gynecologic cancer [142], and outcomes after SBRT for prostate cancer [147]. The occurrence of metabolic syndrome and poor glucose control in obese cancer patients can increase the risk of non-cancer death in survivors [151] and increase the susceptibility to infections including SARS-CoV-2 (COVID-19) [152,153,154,155]. Aerobic and resistance exercise are means to reduce sarcopenia in obese and non-obese cancer patients [145,156]. Concepts of avoiding sarcopenia, reducing metabolic syndrome, and improving function and strength in obese cancer patients, as advocated by Dieli-Conwright and others, can improve the quality of life while on therapy and in cancer survivors [145,148,156,157,158,159,160,161,162,163,164,165]. This involves education to neither lose too much weight nor gain a lot of weight, but to focus on staying active and strong. Aerobic and resistance exercise can improve the problem of sarcopenia and possibly mitigate expectations of worse outcomes in obese cancer patients [145,156,157,158,159,160,161,162,163,164].

Since patient eating behaviors are generally superior as an outpatient compared to inpatient, electronic patient-reported outcomes (ePRO) [69,70,71,72,73,130,137,138,139,140,166,167,168] may help to generate new data and metrics concerning differences between predominantly outpatient versus inpatient chemotherapy delivery and admissions for chemotherapy and amelioration of serious adverse events (SAEs). For example, this approach could ask for (number of hospital days/year) to generate a new ePRO metric of quality and cost of cancer care. This approach could facilitate a quantitative study of variables such as lymphopenia, albumin, glucose control, sarcopenia, and weight loss in relation to clinic days, hospital days, documented infections, disease-free survival, and overall survival.

Quality metrics offer an important on-going means for oncology programs to adapt and adopt the best practices to facilitate better eating during cancer therapy. Prompt responding to PRO data may also contribute positively to the complex and bidirectional nature of reducing chemotherapy and radiation toxicity with concurrent improvements in eating. (Figure 1). PRO may also help cancer patients and caregivers to better sort out major versus minor contributors to eating behavior and well-being as well as staying “on-track” during treatment and avoiding polypharmacy or worse: ineffective, and potentially harmful supplements and unproven alternative treatments [41,74,128,129,130,131,132,169].

To facilitate better short-term and long-term outcomes, the oncology team’s efforts should strive to have reliable and predictable care and to reduce “battle fatigue”. This involves flexible and adaptable scheduling, reducing futile care, and fewer interventions that miss the mark concerning cancer control (Figure 2) in addition to developing therapeutic alliances to continue sustainable efforts (Table 6). This team approach also may use radiation to reduce pain or definitively treat life-limiting metastases with SBRT to reduce both cancer burden and “scanxiety”. A combination of therapeutic alliances to facilitate state-of-the-art cancer treatment with toxicity reduction, better eating behaviors, and improved quantity and quality of nutrient intake can help the patient and oncology team feel they have performed to their best to facilitate an improved cancer outcome.

## 4. Summary and Conclusions

This article reviews the complex and often bidirectional variables associated with chemotherapy and radiation toxicities and their effects on eating behaviors. A proactive and adaptive approach using feedback for toxicity and side-effect amelioration is advocated. In conclusion, developing better therapeutic alliances to reduce chemotherapy and radiation toxicities are important for oncology professionals, others in the medical center, and in the cancer patient’s social and community networks.

## 5. Patents

PM Anderson glutamine and trehalose compositions US2015/0080331 pub date 19 March 2015.

## Figures and Tables

**Table 6:** Therapeutic alliances of cancer patients to obtain information and support.

Oncology Professionals	Others in the Clinic and Hospital	Community Resources
Medical Oncologist	Dietitian (nutritionist)	Primary caregiver
Pediatric Oncologist	Social worker	Other family
Radiation Oncologist	Psychological support (coping skills)	Friends
Oncology Surgeon	Physical therapy (PT)	Peer support (disease-specific groups)
Nurse Educator	Occupational therapy (OT)	Facebook and other internet sites
Oncology Pharmacist	Art therapy	On-line consults
Oncology Navigator	Music therapy	Insurance case manager
Oncology Nurse Practioner	Scheduling	Faith community
Oncology Physician Assistant	Lab (e.g., phlebotomy) personnel	School support
Chemotherapy Nurse Virtual Oncologist [52]	Radiology personnel Quality teams Child life specialists	Employee support Neighbors

## Abbreviations

**Abbreviation**	**Spelling**	**Context and Comment**
SBRT	Sterotactic body radiotherapy	Very precise radiation given in 1–5 treatment sessions
MASCC	Multinational Association for Supportive Care in Cancer	Organization which includes experts that provide guidelines for improving side effects of cancer and cancer treatment
ESMO	European Society of Clinical Oncology	Organization of cancer professionals to share information and guidelines to develop better ways to treat cancer in Europe and other regions. The academic journal is ESMO Open
ASCO	American Society of Clinical Oncology	World’s largest organization of cancer professionals to share information and guidelines to develop better ways to treat cancer. Academic journal is the Journal of Clinical Oncology
NCCN	National Comprehensive Cancer Network	An organization devoted to providing guidelines for treatment of specific cancers and issues related to cancer treatment
PG-SGA	Patient Generated Subjective Global Assessment	A tool to help identify malnutrition and cachexia in cancer patients at risk for sarcopenia
OBD	Optimal biologic dose	Dose of an agent that achieves best effect against a target with acceptable toxicity (for example effect on immune activation)
MTD	Maximally tolerated dose	Dose of a drug (e.g., chemotherapy drug) for which an increase dose would have unacceptable toxicity. Once the MTD is determined in a phase I clinical trial, this becomes the recommended phase 2 dose of an anti-cancer agent
AUC	Area under the curve	A pharmacokinetic/pharmacodynamic concept that reflects the graph of drug concentration over time. Usually, an inverted U shape because of absorption, distribution, and metabolism, and elimination of an agent
VEGF	Vascular endothelial growth factor	A protein involved in generation of new blood vessels to heal wounds or injury or associated with tumors growing new blood vessels. Some anti-cancer drugs block VEGF
NG	Naso-gastric	Usually refers to thin tube that extends from the nose to the stomach to provide liquid nutrition, suspensions of drugs, or fluids without swallowing
G-tube	Gastrostomy tube	A tube that goes directly from abdominal skin through the muscles and lining of the abdomen into the stomach. Same use as NG but without the discomfort of a tube in the nose or back of the throat
SRS	Stereotactic radiosurgery	Ultraprecise radiation that may require a “halo” device or anesthesia to give the dose to tumor only in the brain or near the spinal cord
N/V	Nausea and/or vomiting	The most common side effect of cancer chemotherapy
5HT	5-hydroxytryptamine (serotonin)	The 5-HT3 receptor is triggered in the brain chemoreceptor trigger zone to cause drug associated N/V. Inhibitors of 5-HT are very useful as anti-emetics to reduce or prevent chemotherapy associated N/V
TPN	Total parenteral nutrition	An intravenous solution containing glucose, amino acids, vitamins, and sometimes lipids that is used when patients cannot eat or drink adequate amounts of nutrients
TKI	Tyrosine kinase inhibitors	A class of drugs that act to block cancer-associated tyrosine kinase enzyme(s) in cancer cells that facilitate cancer growth
WART	Whole abdominal radiotherapy	A radiation technique to provide a moderately high dose of radiation to the entire abdomen with relative sparing of liver and kidneys and treating intestines and intestinal lining to tolerance dose.
RT	Radiotherapy	Use of radiation as a treatment modality for cancer
BMT	Bone marrow transplant	High-dose chemotherapy followed by infusion of marrow or blood stem cells to allow recovery of blood cell production
G-CSF	Granulocyte colony stimulating factor	A subcutaneous injection given after chemotherapy to increase granulocyte (neutrophil) production by the bone marrow to make chemotherapy safer
KPS	Karnofsky performance scale	A scale from 0% (dead) to 100% (full activity without limitation) to indicate how active a cancer patient is and whether or not activity (performance) is limited by symptoms of drugs, radiation, or cancer
PT/OT	Physical therapy and/or occupational therapy	PT involves improving function, exercise, and strength training. OT involves learning how to do activities of daily living better (e.g., climbing stairs, opening a jar, buttoning a shirt)
GNRI	Geriatric Nutrition Risk Index	A composite compilation of risk factors to help with malnutritional assessment in older people
MNA	Mini-Nutritional Assessment	A composite compilation of risk factors to help with malnutritional assessment
PRO	Patient reported outcome	A self-assessment form (if electronic it is an ePRO)
BMI	Body mass index	A calculation involving height and weight that can give a number to indicate thin, normal or obese (e.g., BMI > 30)
